# Modeling congenital heart disease: lessons from mice, hPSC-based models, and organoids

**DOI:** 10.1101/gad.349678.122

**Published:** 2022-06-01

**Authors:** Kavitha S. Rao, Vasumathi Kameswaran, Benoit G. Bruneau

**Affiliations:** 1 Gladstone Institutes, San Francisco, California 94158, USA;; 2 Roddenberry Center for Stem Cell Biology and Medicine at Gladstone, San Francisco, California 94158, USA;; 3 Department of Pediatrics and Cardiovascular Research Institute, University of California at San Francisco, San Francisco, California 94158, USA

**Keywords:** congenital heart disease, genetics, heart

## Abstract

Congenital heart defects (CHDs) are among the most common birth defects, but their etiology has long been mysterious. In recent decades, the development of a variety of experimental models has led to a greater understanding of the molecular basis of CHDs. In this review, we contrast mouse models of CHD, which maintain the anatomical arrangement of the heart, and human cellular models of CHD, which are more likely to capture human-specific biology but lack anatomical structure. We also discuss the recent development of cardiac organoids, which are a promising step toward more anatomically informative human models of CHD.

## Abstract (precis)

In this review, Rao et al. contrast mouse models of CHD, which maintain the anatomical arrangement of the heart, and human cellular models of CHD, which are more likely to capture human-specific biology but lack anatomical structure. They also discuss the recent development of cardiac organoids, which are a promising step toward more anatomically informative human models of CHD.

Cardiac development is achieved by the intricately coordinated actions of several transcription factors (TFs) and chromatin modifiers to guide timely activation of specific genes. This cascade of transcriptional events ensures that appropriate cellular and structural cues are relayed to generate a complex, four-chambered structure from the cardiac crescent. Congenital heart defects (CHDs) are malformations of the developing heart, and occur in at least one out of 100 live births (van der Linde et al. 2011). Some are very severe, resulting in neonatal death or requiring complex open heart surgery. Oftentimes, the repair only provides a temporary solution, and the patient requires heart transplantation a few years later. In addition, structural CHDs can be accompanied by physiological defects, such as abnormal impulse conduction or heart failure. Structural defects in CHD patients are often associated with mutations in the gene regulators that play a role in very early cardiogenesis, including TFs, chromatin remodeling factors, and signaling molecules (Nees and Chung 2019; Morton et al. 2022). However, the links between disease-causative variants and the resulting heart defect are not well understood. Therefore, there is a great need for disease modeling to better understand CHD pathophysiology and discover treatment options. Here, we review the current literature on modeling CHDs, exploring the relative benefits and limitations of mouse models and human cellular models.

## Modeling CHDs in mice

The pathogenesis of CHDs is complex. A majority of CHDs lack a clearly identifiable heritable cause (for extensive review, see Nees and Chung 2019; Morton et al. 2022), although this may be attributable to myriad factors, including as yet unidentified genes, lowly penetrant multigenic disorders, or uterine environment. However, human genetics research has begun to unravel the genetic basis in some cases of CHDs, pointing to errors in common cellular mechanisms such as transcriptional regulation. Notably, many CHD variants have been identified in highly conserved cardiac TFs that are central to establishing cardiac morphology and progenitor identity. Apart from a few important exceptions, mouse heart development closely resembles human heart development, so mouse models of CHD can provide an excellent opportunity to understand how specific factors are involved in heart development and contribute to disease development. Here, we review mouse models that have provided insights into CHDs and the common themes that have emerged from disease modeling in mice. We particularly focus on some of the first TFs that were identified as CHD variants due to the extensive body of literature that has shed light on various aspects of their roles in CHD and cardiac development.

### Roles of CHD genes in early cardiogenesis

Patterning of gene expression can provide clues about the potential function of CHD genes. TBX5 heterozygous loss-of-function mutations underlie Holt-Oram syndrome (HOS), an autosomal dominant developmental disorder characterized by skeletal abnormalities and, frequently, cardiac defects including atrial septal defects (ASDs), ventricular septal defects (VSDs), and conduction defects (Basson et al. 1994, 1997; Newbury-Ecob et al. 1996; Li et al. 1997). The pattern of *Tbx5* expression in the developing embryo is consistent with the clinical characteristics of HOS (Chapman et al. 1996; Bruneau et al. 1999). In human embryos, *TBX5* is highly expressed in the developing inflow tract, atrial wall, atrial septa, and atrioventricular (AV) endocardial cushions (Li et al. 1997). In mouse and chick embryos, where it has been characterized more extensively over the course of cardiac development, *Tbx5* exhibits a unique asymmetric pattern of expression. It is initially expressed in the cardiac crescent, but becomes increasingly restricted to the atria and then the left ventricle following looping and septation (Bruneau et al. 1999). In the ventricles, *Tbx5* expression is finely restricted to the boundary between the left and right sides of the interventricular septum (IVS). Indeed, disrupting the location of this boundary by either removing or extending the domain of Tbx5 expression leads to a lack of IVS. Lineage tracing further shows that Tbx5^+^ cells form what appears to be a lineage boundary at the junction of the left and right sides of the IVS (Devine et al. 2014). These data together suggest that VSDs caused by reduced TBX5 function might be due to mispatterning of this IVS boundary.

On the other hand, some genes, such as the cardiogenic TF *Nkx2-5*, are broadly expressed throughout embryonic heart development. In mice, *Nkx2-5* is expressed in the earliest cardiac progenitors in the late primitive streak, before the onset of myogenic differentiation (Komuro and Izumo 1993; Lints et al. 1993). It is abundantly expressed throughout the atrial and ventricular myocardium, but not in the endocardium or epicardium. Heterozygous loss-of-function *NKX2-5* mutations were initially identified in patients with secundum ASDs and AV conduction defects (Schott et al. 1998) Subsequent studies revealed that the spectrum of CHDs caused by *NKX2-5* mutations is somewhat broader than initially recognized (Benson et al. 1999), but indicated that although the factor is widely expressed, only some regions of the heart are sensitive to reduced NKX2-5 function, and there is variation in which regions develop structural defects in its absence. This suggests that other spatially restricted cues might interact with NKX2-5 to confer dosage sensitivity.

During early cardiac development, the GATA transcription factor family homologs *Gata4*, *Gata5*, and *Gata6* share very similar expression patterns, but they have overlapping yet distinct domains of expression during later stages (Arceci et al. 1993; Laverriere et al. 1994; Morrisey et al. 1996), suggesting that they may each contribute to fine spatial and temporal regulation differences in cardiac development. In contrast to GATA4 and GATA6, which are expressed broadly in the primitive streak mesoderm (Heikinheimo et al. 1994; Morrisey et al. 1996; Koutsourakis et al. 1999), GATA5 has a more restricted expression pattern in the precardiac mesoderm (Morrisey et al. 1997). GATA4 and GATA6 have overlapping expression patterns in the developing myocardium, vascular smooth muscles, and endocardial cushions (Heikinheimo et al. 1994; Morrisey et al. 1996; Koutsourakis et al. 1999), whereas GATA5 expression becomes progressively restricted to the atrial endocardial cells before its cardiac expression generally diminishes (Morrisey et al. 1997). GATA6 is highly expressed in the cardiac outflow tract (OFT) and neural crest cells that are required for proper OFT development (Lepore et al. 2006). GATA6 is also detected throughout the sinoatrial node by E14.5 in mice (Gharibeh et al. 2021) and, accordingly, cardiac conduction defects are observed in individuals with mutations in *GATA6*, whereas *GATA4* mutations more often result in septal defects and endocardial cushion defects, but not issues with conduction (Garg et al. 2003). While compensation and genetic interaction of these factors have been studied in mouse knockout models, the extent to which each gene can compensate for the others in human CHDs is yet to be determined.

### Haploinsufficiency in mouse models vs. human CHD

Virtually all genetically defined CHDs are due to reduced gene function, whether it be because of a gene deletion, nonsense mutation, or loss-of-function missense mutation. This indicates that a common molecular mechanism underlies many CHDs: exquisite sensitivity to the dosage of these proteins. Although humans have clear and often severe CHDs in the context of haploinsufficiency of the causative genes, in mice this is highly variable.

Both mice and humans display sensitivity to *Tbx5* dosage (Bruneau et al. 2001; Mori et al. 2006; Moskowitz et al. 2007). Indeed, haploinsufficiency of *Tbx5* in mice reasonably phenocopies the hallmarks of HOS, including the asymmetric bilateral upper limb, frequent ASDs and VSDs, and abnormal cardiac electrophysiology, possibly due to poor formation of the conduction system (Bruneau et al. 2001; Moskowitz et al. 2007), as well as impaired cardiac relaxation (Zhu et al. 2008). Notably, *Tbx5* heterozygous null mice are not recovered at Mendelian ratios at birth, indicating perinatal lethality, and those that die in utero exhibit a complex range of cardiac defects including ASDs, muscular VSDs, and deformed left ventricles (Bruneau et al. 2001). Using mouse models with a gradation of *Tbx5* dosage, Mori et al. (2006) found that the phenotype, including severity of ASDs, paralleled the expression level of *Tbx5* regardless of genetic background. Changes in gene expression were also sensitive to Tbx5 dosage (Mori et al. 2006). However, humans with *TBX5* mutations also have other cardiac defects including tetralogy of Fallot (ToF), and rarely hypoplastic left heart syndrome (HLHS) (Basson et al. 1994; Newbury-Ecob et al. 1996), but these defects are not observed in *Tbx5* heterozygous mice.

Mutations in *NKX2-5* have been identified in families with a high incidence of secundum ASDs and high penetrance of AV conduction defects (Benson et al. 1998; Schott et al. 1998). Affected individuals also have other structural abnormalities including VSDs, ToF, aortic stenosis, left ventricular noncompaction syndrome (LVNC), HLHS, and dilated cardiomyopathy (Schott et al. 1998; Elliott et al. 2003; Sarkozy et al. 2005; Costa et al. 2013). Initial studies examining the effects of *Nkx2-5* loss of function in mice found that complete loss of *Nkx2-5* resulted in embryonic lethality due to the inability of the heart tube to undergo rightward looping despite establishing proper myogenesis (Lyons et al. 1995). Surprisingly, mice that were heterozygous for a *Nkx2-5*-null allele were viable and seemingly normal. However, upon closer examination, it was determined that *Nkx2-5* haploinsufficiency did in fact result in a low frequency of ASDs and an increased incidence of patent foramen ovale (PFO) (Biben et al. 2000; Tanaka et al. 2002). Mice with heterozygous loss of *Nkx2-5* also exhibited mild conduction defects, with prolonged PR and QR intervals and extended atrial fibrillation (Tanaka et al. 2002), which was shown to be related to a change in the number of myocytes that become committed to the conduction system (Jay et al. 2004).

Despite the largely redundant expression patterns of the three cardiac-enriched GATA TFs (Laverriere et al. 1994), haploinsufficiency of the individual factors results in similar, yet distinct, phenotypes. Mouse embryos lacking *Gata6* have defects in extraembryonic tissue development and die shortly after gastrulation (Koutsourakis et al. 1999), while its haploinsufficiency leads to defects in OFT septation and cardiac conduction (Lepore et al. 2006; Gharibeh et al. 2018, 2021), reflective of its expression pattern in these regions during development. Accordingly, human mutations in *GATA6* have been identified in cases of patent ductus arteriosus (PDA) (Kodo et al. 2009) and atrial fibrillation (Yang et al. 2012; Gharibeh et al. 2021).

On the other hand, complete *Gata4* loss of function, while also embryonic lethal, does permit development of cardiac lineage-committed progenitor cells in mice (Kuo et al. 1997; Molkentin et al. 1997). Interestingly, *Gata6* expression was found to be increased in the absence of *Gata4*, suggesting at least a partial functional compensation between these two factors. In fact, loss of both factors resulted in acardia with a block in the progressive differentiation of cardiac progenitors to myocytes (Zhao et al. 2008). Although *Gata4* heterozygous mice were initially believed to undergo normal cardiac development, a study of a gradation of GATA4 protein levels using various hypomorphic alleles revealed that small differences in *Gata4* expression could profoundly affect embryonic viability (Pu et al. 2004). Whereas mice with hypomorphic alleles with 50% of normal GATA4 protein expression levels survived, a further reduction of 20% GATA4 expression resulted in embryonic death between E13.5 and E16.5. Thus, in mice there is a narrow window of tolerance for reduced *Gata4* dosage, below which cardiomyocyte replication, endocardial cushion development, and proper chamber septation are compromised (Pu et al. 2004; Rajagopal et al. 2007). In humans, GATA4 mutations have been identified in two unrelated families with a history of CHDs. Two reported mutations in this study resulted in an autosomal dominant pattern of inheritance in which all affected individuals had ASDs (Garg et al. 2003). However, similar to other TFs implicated in CHDs, these mutations also resulted in a range of CHD phenotypes, including other septal defects and pulmonary valve thickening, but not cardiac conduction abnormalities. Other human mutations have also been identified in *GATA4*, some of which coincide with reported mouse haploinsufficiency phenotypes such as endocardial cushion defects, hypoplastic right ventricle, and secundum ASDs (Rajagopal et al. 2007). However, highly penetrant mouse phenotypes such as cardiomyopathy were not identified in the human patients.

These observations underscore the difference between mice and humans depending on the mutated gene. In addition, they reveal that physiological parameters such as the conduction system may be particularly sensitive to TF dosage and thus more likely to be recapitulated in mouse models than structural defects. Mouse models of these gene deletions have improved our understanding of the functional consequences of haploinsufficiency and how they might relate to disease phenotypes. However, mechanistic insight into how haploinsufficiency of a given TF affects the regulation of its direct targets is still an area of immense interest. Genomics-based approaches to interrogate the dose-dependent effects of haploinsufficiency are proving to be helpful in parsing these effects; however, mouse models can be limiting due to the challenges with generating sufficient material for such experiments.

### Variability in phenotype

Aside from the primary clinical characteristics presented in each family, a wide range of other cardiac phenotypes are also frequently noted in CHD studies (Basson et al. 1994; Newbury-Ecob et al. 1996; Benson et al. 1998; Schott et al. 1998; Brassington et al. 2003; Elliott et al. 2003; Costa et al. 2013). There is variability in phenotype within families that share a common mutation, and this variability extends to the corresponding mouse knockout phenotype for each gene. For example, even mutations that are predicted to result in loss of function of TBX5 exhibit an array of phenotypes within the same family (Brassington et al. 2003).

One explanation for this variability is the contribution of genetic modifiers. Indeed, the remarkable concordance in clinical characteristics of monozygotic twins with a *TBX5* mutation resulting in HOS argues for the direct effect of genotype on disease manifestation (Huang et al. 2002). In mice, this is evidenced by the fact that different strains of mice exhibit varying degrees of severity of their phenotype (Biben et al. 2000; Bruneau et al. 2001; Mori et al. 2006). This is also apparent in mouse models of *Gata4* loss of function, where the genetic strain influences the degree of penetrance of cardiac abnormalities and the perinatal survival rates (Bisping et al. 2006; Rajagopal et al. 2007).

The combinatorial effect of multiple variants was nicely exemplified in a study of LVNC cardiomyopathy by Gifford et al. (2019), who used exome sequencing in a family with high penetrance of LVNC to discover damaging mutations in *MYH7* and the TF *MKL2* inherited from an affected father. However, the additional inheritance of a rare missense variant in *NKX2-5* from their unaffected mother resulted in the three offspring having profoundly more severe disease and earlier age of onset. These conclusions were supported by modeling in mice, which showed that compound heterozygotes for all three genes exhibited LVNC-like phenotypes with hypertrabeculations in the left ventricular wall.

However, the fact that littermates within inbred lines can also show a wide range of variability suggests that genetic modifiers are not the only contributor to phenotypic variability. The discrepancy between human and mouse phenotypes in some cases may be attributable to the fact that the mutations are typically identified in families with a high incidence of disease. Thus, familial carriers of mutations with low disease severity are less likely to be identified, causing a skewed interpretation of the spectrum of phenotypes that these mutations might cause in humans.

### Cooperativity of genes

Proper cardiac development is achieved through the convergent actions of multiple factors across different cell types. Through various studies of human and mouse genetics, a common theme that has emerged is that mutations in a single gene can result in a wide range of cardiac defects and, conversely, that mutations in multiple genes can result in very similar cardiac abnormalities. This is exemplified by the case of *TBX5* and *NKX2-5*, as mutations of each result in ASDs, conduction defects, and other similar cardiac abnormalities. A physical interaction between these two factors was found to synergistically activate expression of common target genes such as *ANF*, *Cx40*, *Nppa*, and *Id2* (Bruneau et al. 2001; Hiroi et al. 2001; Moskowitz et al. 2007). In fact, heterozygous loss of both *Tbx5* and *Nkx2-5* results in the lack of specification of the ventricular cardiac conduction system altogether (Moskowitz et al. 2007). *Nkx2-5* was also found to interact with *Gata4*, a factor with which it shares a common expression pattern from early cardiogenesis (Durocher et al. 1997). A similar cooperative interaction between *Gata4* and *Tbx5* was identified and found to be sensitive to some patient variants of *GATA4*, suggesting one possible mechanism of action by which the mutations might affect downstream cardiac development (Garg et al. 2003).

Recent evidence has also drawn attention to the role of chromatin modifiers and epigenetic regulators in the regulation of cardiac development (Zaidi et al. 2013; Homsy et al. 2015). In fact, variants in several chromatin modifiers have been implicated in CHDs, and mouse models continue to shed light on the mechanisms by which mutations in these ubiquitous transcriptional regulators can result in tissue-specific abnormalities (Zaidi and Brueckner 2017). One mechanism by which they affect cardiac development is through cooperativity with TFs. For example, *Smarcd3*, encoding BAF60C, a subunit of the SWI/SNF-like BAF complex, is expressed specifically in the heart during early mouse development, and its loss results in severe cardiac abnormalities, including defects in development of the OFT and trabeculae of the myocardium (Lickert et al. 2004). *Baf60c* mediates the interaction between cardiac TFs like *Tbx5*, *Nkx2*-5, *Gata4*, and the core ATPase of the BAF complex, *Brg1*, as well as the Mef2 cofactor Myocardin (MYOCD) (Takeuchi et al. 2011; Sun et al. 2017). *Brg1* itself displays strong dosage sensitivity in heart development that is further exacerbated by the compound loss of TFs *Tbx5, Nkx2-5*, and *Tbx20* (Takeuchi et al. 2011).

**Figure 1.**
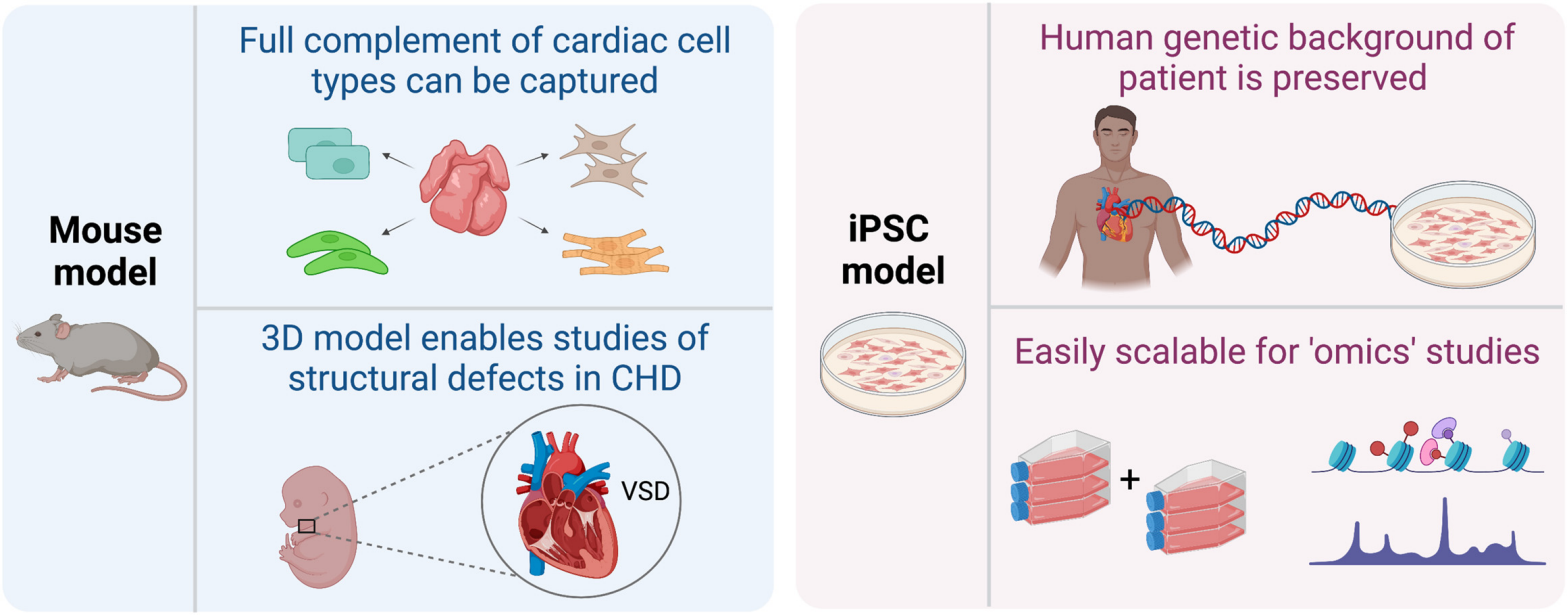
Advantages of mouse and iPSC-based models of CHD. Mouse models of CHDs enable investigation of the various cell types found in the heart that otherwise may not be generated using in vitro differentiation methods. Mouse models also provide spatial context, which is key to recapitulate structural defects found in humans. On the other hand, iPSC-based models allow studies to be carried out in the context of human genetic background and lend themselves to large-scale high-throughput assays. (Created with Biorender.com.)

Overall, mouse models have provided tremendous understanding into the molecular, structural, and phenotypic aspects of the contribution of these genes. However, often the consequences of heterozygous mutations in human CHD genes are not recapitulated in mouse models. This limitation, as well as challenges with phenotypic variability, argue for the complementary use of in vitro models of disease in which some of these issues can be circumvented. The combined insight from these approaches is likely to facilitate the discovery of better treatment options for CHD patients (Fig. 1).

## Modeling CHDs in human iPSC-based systems

The combination of human pluripotent stem cells (hPSCs) and genome-editing tools has revolutionized human disease modeling. The isolation of human embryonic stem cells (hESCs) from human blastocysts (Thomson et al. 1998) and subsequent differentiation to cardiomyocytes (Kehat et al. 2001; Xu et al. 2002; He et al. 2003) opened the doors to cardiac disease modeling in human cells. More recently, the generation of induced pluripotent stem cells (iPSCs) from human adult somatic cells (Takahashi et al. 2007) has expanded the access to patient-specific cells for CHD studies. Genome-editing tools such as CRISPR have enabled the generation of hPSC lines harboring disease-relevant heterozygous or homozygous loss-of-function alleles (Hendriks et al. 2020). There are two major approaches to modeling CHD with these tools. The first involves reprogramming of somatic cells from CHD patients and healthy controls to generate iPSC lines, or isolation of patient iPSCs and the correction of the suspected mutation to create isogenic control lines. Alternatively, CRISPR technology can be used to introduce disease-causing mutations in publicly available “normal” iPSC lines such as WTC11 (Lee et al. 2009) or PGP1 (Miyaoka et al. 2014) and hESC lines such as H9 (Thomson et al.1998). These modified hPSC lines can be further differentiated into various cardiac cell types in vitro (Lian et al. 2012, 2013; Burridge et al. 2014) and used to study the mechanisms underlying cellular defects occurring in CHDs. In the following sections, we focus on a few hPSC-based CHD models to highlight the benefits of this system.

### Controlling for variations in genetic background

CHD phenotypes in humans are often not recapitulated in mouse models, perhaps due to the differences between the human and mouse genomes. Modeling CHDs in hPSC-derived cells is an advantage because the human genetic background is preserved. However, in the case of patient-derived iPSC lines, variation in genetic background between patients and controls persists as a confounding factor. This issue can be circumvented by using CRISPR to correct the disease-causing mutation in the patient-derived iPSCs to generate isogenic control iPSC lines (Theodoris et al. 2015; Ang et al. 2016). Another approach is to show a direct link between the missing gene and CHD phenotype by introducing the candidate mutation via gene editing (Wang et al. 2014).

### Mechanistic insights from iPSC-based CHD models

Cell types derived from diseased iPSCs often recapitulate phenotypes observed in patients, and therefore can provide invaluable insight into disease mechanisms. Despite growing in a dish, iPSC-derived cells are readily amenable to dissection of cellular pathways underlying CHDs via a wide variety of interventions including siRNA knockdown, treatment with inhibitory molecules, biochemical assays, and imaging modalities.

These advantages are exemplified by studies of Barth syndrome (BTHS) in iPSC-derived models. BTHS is a rare X-linked multisystem disorder encompassing dilated cardiomyopathy, skeletal muscle myopathy, growth delay, and neutropenia (Clarke et al. 2013; Saric et al. 2016). Studies of BTHS in human cellular models, including patient-derived fibroblasts (Barth et al. 1996) and lymphoblasts (Gonzalvez et al. 2008), were restricted by the limited supply of patient cells. Therefore, in order to study cardiomyocyte-specific disease pathogenesis in BTHS, Wang et al. (2014) generated two independent iPSC lines from unrelated Barth syndrome patients, which were then differentiated into cardiomyocytes (CMs) in vitro. BTHS is caused by mutations in the *TAZ* gene (Barth et al. 1983; Bione et al. 1996), which encodes tafazzin, an acyltransferase required for the acylation of a mitochondrial membrane phospholipid called cardiolipin (Neuwald 1997; Vreken et al. 2000). The patient-derived iPSC-CMs (referred to as BTHS iPSC-CMs) recapitulated a hallmark of BTHS: elevated ratios of monolysocardiolipin and mature cardiolipin. As BTHS is a metabolic disorder, the investigators leveraged various cell-based assays to demonstrate that BTHS iPSC-CMs displayed reduced ATP levels, higher oxygen consumption rates, and diminished respiratory capacity. The mitochondrial dysfunction in BTHS iPSC-CMs was further linked to irregularity in sarcomere spacing. The cardiomyopathy often observed in BTHS patients was reflected in the poor contractility of BTHS iPSC-CMs. Interestingly, *TAZ* gene replacement in BTHS iPSC-CMs returned cardiolipin levels to near normal and restored mitochondrial function, suggesting the potential for gene replacement therapy in the treatment of BTHS. Indeed, adenovirus-mediated *TAZ* gene replacement therapy in *TAZ* knockout mice led to reversal of cardiac dysfunction (Wang et al. 2020). These studies showcase the power of human iPSC-based models of CHD to elucidate disease mechanisms and serve as a preclinical testing ground for potential therapeutic approaches.

### Scalability of hPSC-based models for ‘omics’ studies in CHDs

As discussed above, many CHDs are caused by mutations in early cardiac TFs such as *GATA4*, *TBX5*, and *NKX2-5*, which steer broad gene expression programs leading to changes in cell identity. Understanding how reduction in TF dosage can alter the transcriptional and epigenomic landscape requires multiomic approaches, such as single-cell RNA-seq, ChIP-seq, and ATAC-seq. Although cardiovascular cells from CHD patients are typically inaccessible for such studies, iPSC-derived cells are a renewable source. In vitro differentiation methods allow researchers to produce millions of diseased cells from hPSCs with relative ease, facilitating such large-scale assays.

A powerful example of such an approach involves the study of the G296S missense mutation in *GATA4*, which has been linked to fully penetrant atrial or ventricular septal defects, atrioventricular septal defects, and pulmonary stenosis (Garg et al. 2003). To study the effects of this mutation in human cells, Ang et al. (2016) generated iPSCs from four patients harboring G296S. Following cardiac differentiation, G296S CMs displayed defective calcium transients, disorganized sarcomeres, and decreased contractile force, mimicking the cardiomyopathy observed in patients. RNA-seq analysis in mutant iPSCs undergoing CM differentiation highlighted a broad down-regulation of the cardiac gene program, including genes involved in the Hedgehog signaling pathway, cardiac chamber morphogenesis, heart contraction, myofibril assembly, and cardiac progenitor differentiation. Instead, mutant cells showed an up-regulation of genes linked to the endocardial/endothelial development, with a concomitant increase in ATAC-seq signal at these sites, suggesting incomplete silencing of alternative gene programs during cardiac differentiation. In addition, the investigators characterized superenhancers (SEs)—large clusters of enhancers that are critical in controlling gene expression programs that drive cellular identity changes (Whyte et al. 2013). In G296S cells, TBX5 recruitment to SEs was reduced, especially near key cardiac genes such as *RBM20*, *SMYD1*, and *SRF*, suggesting that impaired GATA4–TBX5 interaction in mutant cells was a driving force in the mutant phenotype.

To explore the role of *NKX2-5* in human cardiac development, Anderson et al. (2018) established a *NKX2-5^−/−^* human embryonic stem cell (hESC)-based model. Although *NKX2-5^−/−^* hESCs formed beating CMs, they lacked VCAM1, a marker of cardiac fate commitment, and sustained expression of PDGFRa, a cardiac progenitor marker, indicating stalled differentiation. *NKX2-5^−/−^* CMs displayed reduced asynchronous beating with a prolonged action potential duration, as well as reduced contractile force. A combination of gene expression profiling and NKX2-5 chromatin occupancy analysis revealed that the absence of NKX2-5 results in down-regulation of markers of ventricular development such as *IRX4*, *HAND1*, *HEY2*, and *MYL2* and up-regulation of smooth muscle markers including *MYH11* and *TAGLN*. Moreover, temporal expression of *HEY2* in *NKX2-5^−/−^* CMs leads to restoration of *VCAM1* expression and repression of *MYH11* expression, thus implicating HEY2 as one of the mediators of NKX2-5 function.

Understanding how TF haploinsufficiency may lead to CHD requires identification of genes sensitive to TF dosage in a quantitative manner. As cardiac TFs may orchestrate the expression of a multitude of interconnected genes, building gene regulatory networks can be beneficial in this context. To study the effects of TBX5 dosage during human cardiac differentiation, our group developed a human iPSC-based allelic series consisting of heterozygous (*TBX5^in/^*^+^) and homozygous (*TBX5^in/del^*) mutant iPSC lines (Kathiriya et al. 2021). In this case, a mutation in *TBX5* was introduced in a well-defined iPSC line, WTC11, by genome editing. As opposed to correcting a patient mutation, this approach does not carry the genetic background of a patient, which allows determination of the effect of a single introduced mutation on cellular phenotype. Single-cell RNA sequencing showed dysregulation of cardiac development genes, electrophysiology-related genes, and numerous human CHD genes, many of which changed in a stepwise fashion from wild type (WT) to *TBX5^in/^*^+^ to *TBX5^in/del^* in a TBX5 dose-dependent manner. Gene regulatory network analysis highlighted a human cardiac gene network controlled by *TBX5*, with changes in biological importance or “pagerank” of several genes in *TBX5^in/^*^+^ and *TBX5^in/del^* cells. The gene that showed the highest change in pagerank was *MEF2C*, and compound heterozygotes of *Tbx5* and *Mef2c* in mice displayed ventricular septal defects. This study highlights the utility of human TF gene networks discovered in a two-dimensional iPSC model in uncovering genetic interactions underlying structural defects in an in vivo model.

In humans, the majority of mutations in *GATA6* result in OFT abnormalities (Kodo et al. 2009). In a human iPSC-based model of *GATA6* mutations, Sharma et al. (2020) used single-cell transcriptomics to show down-regulated expression of genes critical for OFT development, such as *SMYD1*, *HAND2*, and *KDR*. On the other hand, increased expression of fibroblast markers, epithelial-to-mesenchymal transition markers, and neural development genes indicated that *GATA6*^+/*−*^ iPSCs may adopt alternative fates in the absence of normal cardiac signaling. Analysis of ChIP-seq and ATAC-seq data in WT cells showed 88% of GATA-bound peaks overlapped with closed chromatin, suggesting that GATA6 acts as a pioneer cardiac factor. Unlike open chromatin regions, GATA-bound peaks in closed chromatin regions were sensitive to reduction in GATA6 dosage in *GATA6*^+/*−*^ cells.

Overall, these studies illustrate the potential for discovery of gene regulatory networks that might be disrupted in human CHDs. They also demonstrate the utility of in vitro iPSC models to understand physiological parameters that are independent of the structural defects found in patients.

### iPSC-based disease models for drug discovery: *NOTCH1* story

**Figure 2.**
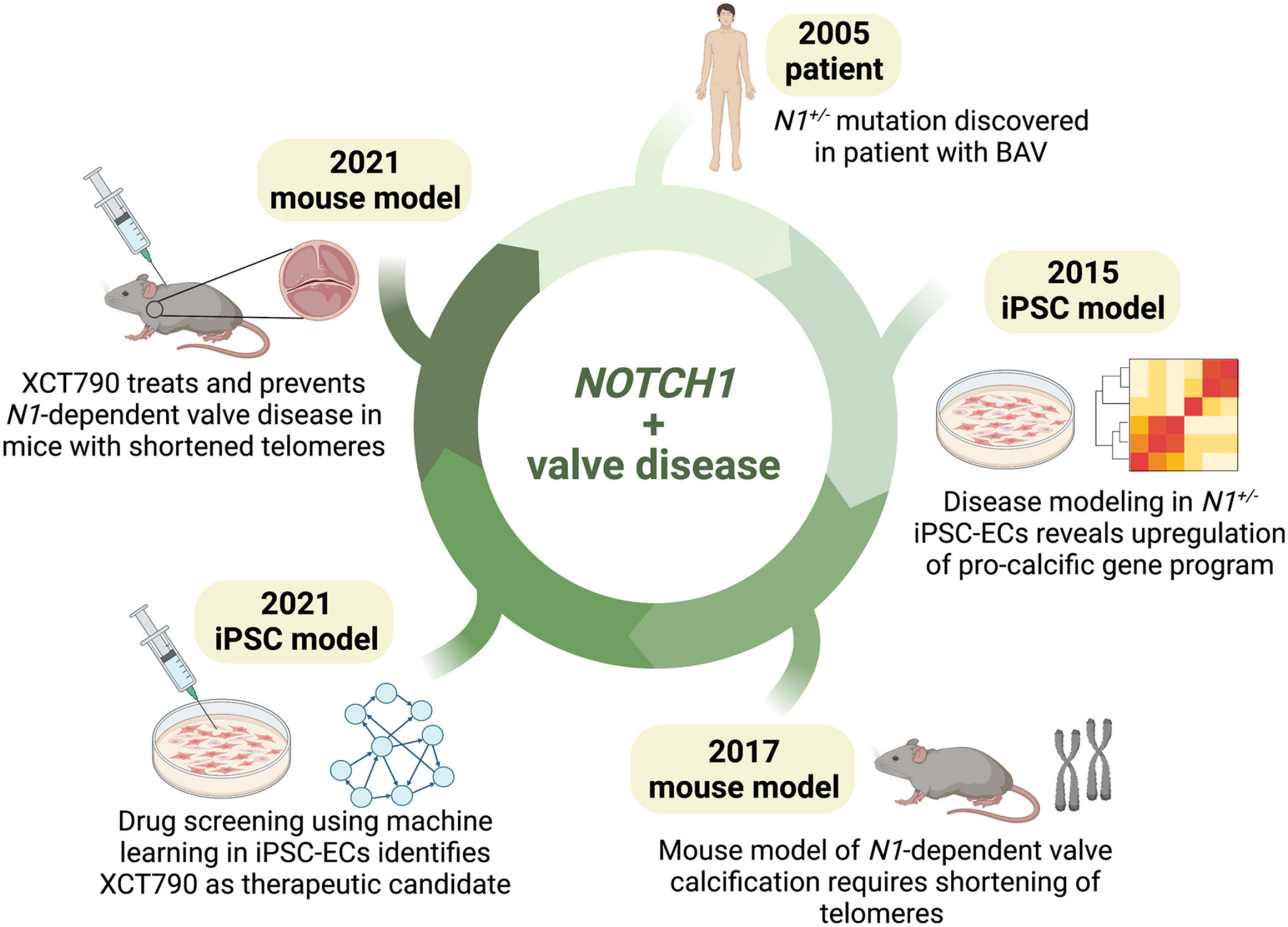
From bedside to bench and back: contributions of mouse and iPSC-based models to *NOTCH1*-related valve disease and drug development. A summary of discoveries made in mouse and human iPSC-based models demonstrates how contributions from both models have come full circle, leading to the development of therapy for valve disease. Since the discovery of *N1^+/−^* mutation in BAV patients, *N1*-related valve disease has been modeled in iPSC-derived endothelial cells (ECs), which demonstrated up-regulation of a procalcific gene program using single-cell transcriptomics. Modeling *N1*-dependent valve disease required shortening of telomeres, highlighting why some mouse models may not recapitulate human CHD phenotypes. A drug screen in the iPSC-based model was then used to discover XCT790 as a therapeutic candidate, which corrected aberrant gene networks in *N1^+/−^* ECs and treated valve calcification in the mouse model. (Created with Biorender.com.)

iPSC-derived cells allow high-throughput screening of candidate small molecules that can rescue disease phenotypes. Moreover, mass production of specific cell types from iPSCs enables drug screening to be performed in disease-relevant cell types. Hence, they serve as a promising preclinical platform to discover lifesaving drugs, which is especially beneficial for CHDs, where expensive surgeries are often the only available therapeutic intervention. Here, we discuss the contributions of iPSC models to the development of therapeutic drug candidates for valve disease (Fig. 2).

Heterozygous *NOTCH1* mutations cause bicuspid aortic valve (BAV), a congenital heart defect that affects 1%–2% of the adult population (Garg et al. 2005). Mutations in *NOTCH1* also lead to calcific aortic valve disease (CAVD) in humans (Garg et al. 2005), which is the most prevalent heart valve disease worldwide with no available therapies other than surgical valve replacement. Patient tissue samples are only available from individuals with severe aortic valve calcification, hindering studies of early disease progression. Hence, there was a pressing need for a model of *NOTCH1* haploinsufficiency for mechanistic studies of CAVD.

Previous studies of valve calcification in mice implicated NOTCH1 signaling in endothelial cells (ECs) (Hofmann et al. 2012). Therefore, Theodoris et al. (2015) set out to establish a human iPSC-derived EC model of CAVD. To this end, they generated iPSCs from CAVD patients with heterozygous *NOTCH1* mutations (*N1^+/−^*) and then differentiated them into ECs (Theodoris et al. 2015). To mimic the hemodynamic shear stress experienced by valves in the heart, *N1^+/−^* iPSC-ECs were subjected to shear stress in vitro. Transcriptomic profiling of WT iPSC-derived ECs subjected to shear stress showed activation of antiosteogenic and anti-inflammatory gene programs, whereas shear-stressed *N1^+/−^* ECs up-regulated pro-osteogenic genes such as *BMP4* and proinflammatory genes such as *IRF6*. Furthermore, genes dysregulated in *N1^+/−^* ECs were associated with lower levels of bound N1, suggesting sensitivity to TF haploinsufficiency. Combining transcriptomic and epigenetic data in *N1^+/−^* ECs, a gene regulatory network was constructed, identifying several key downstream nodes, including procalcific genes *SOX7* and *TCF4*.

Meanwhile, in mice, Theodoris et al. (2017) found that *Notch1* heterozygosity did not lead to valve disease. Reasoning that calcification is an age-dependent phenomenon, these investigators bred the *Notch1* heterozygous mice to mice lacking telomerase activity. Progressively shortening the length of the telomeres in *Notch1* heterozygous mice caused valve stenosis, demonstrating that longer telomeres in mice may slow the progression of age-dependent valve disease. This provided a long-sought mouse model of valve calcification.

Next, Theodoris et al. (2020) used the *N1^+/−^* iPSC-derived EC model to perform a network-based drug screen. Wild-type or *N1^+/−^* ECs were treated with 1595 small molecules, followed by targeted RNA-seq of 119 genes, consisting of a subset of nodes identified from their previous *NOTCH1* gene network (Theodoris et al. 2020). The investigators trained a machine learning algorithm to classify the gene networks of untreated wild-type and *N1^+/−^* ECs with 99.3% accuracy. When they used networks from treated *N1^+/−^* ECs as input, the algorithm identified 11 small molecules that corrected the aberrant *N1^+/−^* EC network to wild-type-like. Of these compounds, XCT790 corrected the most numbers of genes, including central players like *SOX7* and *TCF4*. XCT790 was also effective in correcting the gene networks of primary valve ECs from patients with sporadic valve calcification in the absence of *N1* haploinsufficiency, emphasizing XCT790 as a generalizable treatment for all forms of CAVD. Finally, the investigators tested the ability of XCT790 to correct gene networks in vivo in *Notch1^+/−^/mTR^G2^* mice with valve calcification. Indeed, XCT790 not only restored gene networks in *Notch1^+/−^/mTR^G2^* mice, but also significantly reduced aortic and pulmonary valve thickness in mice with valve disease. *Notch1^+/−^/mTR^G2^* mice treated with XCT790 displayed a reduced risk of developing valve calcification, highlighting the use of XCT790 as a preventative medicine that could delay the onset of valve disease. The strength of this study lies in the strategy of using a gene network-level readout to test the efficacy of a drug molecule, rather than focusing on a limited number of genes or phenotypes. While bicuspid valves are irreversible, the use of XCT790 as a preventative and corrective therapy for valve calcification is highly promising and may reduce the clinical burden of valve replacements worldwide.

The series of experiments leading to the discovery of a potential therapeutic for CAVD illustrates the power of human iPSC-based modeling when combined with bioengineering and transcriptional and epigenomic analyses.

## Modeling in 3D: the promise of cardiac organoids

**Figure 3.**
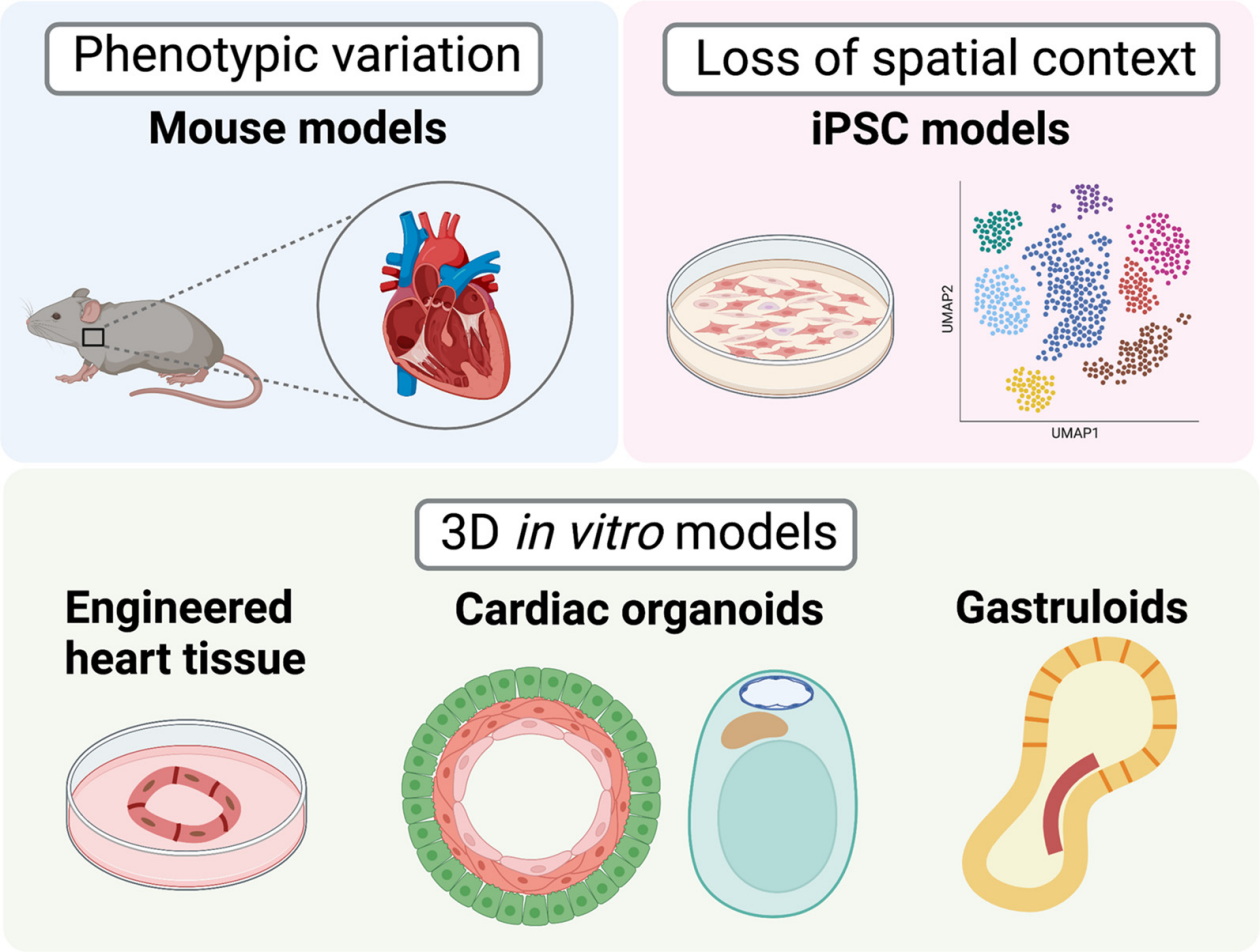
The promise of 3D in vitro models of CHDs. The disadvantages of mouse models include phenotypic variability from human disease. On the other hand, iPSC-based models suffer from the lack of spatial context. Engineered heart tissue, cardiac organoids, and gastruloids hold the promise of overcoming these limitations. (Created with Biorender.com.)

Building on success in self-patterning of mouse ESCs into “gastruloids” with patterned early cardiac structures (Rossi et al. 2021), several groups have developed differentiating iPSCs cultured into three-dimensional (3D) structures that could be referred to as cardiac organoids. These have the potential to act as human organ-like models for the study of CHDs (Fig. 3).

In an approach that used hESCs encapsulated in Matrigel and provided cardiac differentiation cues (e.g., CHIR), Drakhlis et al. (2021) were able to reproducibly derive patterned structures consisting of a noncardiac inner core surrounded by a layer of NKX2-5 and cTnT^+^ CMs. Immunostaining showed the presence of endothelial cells arranged in what appeared to be vasculature. An additional feature was the presence of foregut-like structures adjacent to the cardiac tissue. The CMs could beat and had electrophysiological properties similar to ventricular CMs. When the investigators used *NKX2-5*-null ESCs, the cardiac component was larger and exhibited disorganized sarcomeres and altered gene expression. These phenotypes were somewhat reminiscent of the effect of loss of *Nkx2-5* in mice but did not indicate how loss of *NKX2-5* might alter gene regulation in CHDs.

Drawing from known signaling pathways in vivo and established human pluripotent stem cell differentiation protocols (Kattman et al. 2011), Hofbauer et al. (2021) found that by withdrawing exogenous ECM and finely tuning BMP and WNT signaling, hESCs and iPSCs would reproducibly form beating spherical arrangements that they referred to as “cardioids.” The cardioid model has an interesting feature in that it has what resembles a chamber. Unlike normal heart development, however, the human cardioid “chamber” forms by cavitation. Nonetheless, it has features of a cardiac chamber in that its cellular arrangements are patterned from the inside out. Furthermore, depending on growth factors added, an endocardial lining could be visualized. In a proof of principle experiment, homozygous deletion of the chamber-promoting gene HAND1 led to a failure of cavitation, suggesting that this structure could be used to model CHDs that affect chamber formation.

A very different model involves the spontaneous coemergence of cardiac and gut tissue (Silva et al. 2021). Surrounded by a TBX18^+^ lining reminiscent of epicardium, these very large organoids develop a central core of beating myocardium, a vasculature, and structures that highly resemble intestinal tissue. Based on single-cell RNA-seq, a classifier trained on human fetal heart data predicted that the CMs in these organoids are atrial. This is an unusual finding, as most cardiac differentiation protocols yield mostly ventricular cardiomyocytes (Lee et al. 2017; Hofbauer et al. 2021; Kathiriya et al. 2021). The resulting CMs also had more mature phenotypes than those that arise in most two-dimensional cultures.

## Limitations of CHD models

We have compared and contrasted the model systems used for studying CHDs and described major findings derived from each approach. No single model is a replacement for the others, and the insights from each model should be considered complementary toward our understanding of CHDs.

Mouse models faithfully represent many aspects of human cardiac development, and thus provide incredible insight into the role of specific genes and cell types that contribute to proper cardiogenesis. Despite the advantages that it presents, mutations modeled in mice often do not recapitulate many aspects of the human phenotype. While it is not always apparent what the underlying reasons for these discrepancies might be, additional genetic and environmental variants not captured by individual gene mutations might partially contribute to these differences. Thus, using hPSCs derived from patients may provide insight into these complex mechanisms.

While hPSC-based CHD models are highly scalable human models, there are currently no examples of CHDs that can be modeled only in hPSC-derived cells but not in mice. Rapidly evolving single-cell multiomics technologies now allow efficient capture of cell types from mouse hearts for genomics studies. Moreover, hPSC-based models are two-dimensional and lack the spatial context of the human heart, which is critical for studying structural defects of CHDs. On the other hand, spatial transcriptomics technologies are now facilitating transcriptomics assays in the heart while preserving spatial anatomy (Asp et al. 2019; Mantri et al. 2021).

Although cardiac organoids hold great promise as 3D models of CHD, some aspects of heart physiology such as blood flow cannot be mimicked in a dish. While there are only a few demonstrated examples of cardiac organoids, and they do not fully recapitulate cardiac anatomy, they illustrate that it is possible to generate 3D human structures.
